# Diacetonitrile­(3-{2-[8-(2-bromo­eth­oxy)-9,10-dioxoanthracen-1-yl­oxy]eth­yl}-1-(2-pyridyl­meth­yl)imidazolium)silver(I) bis­(hexa­fluorido­phosphate)

**DOI:** 10.1107/S1600536810029739

**Published:** 2010-07-31

**Authors:** Qing-Song Wen, Cheng-Lin Zhou, Da-Bin Qin

**Affiliations:** a School of Chemistry and Chemical Engineering, China West Normal University, Nanchong 637002, People’s Republic of China

## Abstract

The title compound, [Ag(C_27_H_23_BrN_3_O_4_)(CH_3_CN)_2_](PF_6_)_2_, is a mononuclear salt species in which the silver(I) atom is coordinated by one ligand and two acetonitrile mol­ecules and exhibits a distorted T-shaped coordination. The asymmetric unit contains one independent cation and two independent hexa­fluorido­phosphate anions, one of which is disordered over two positions in a 0.756 (11):0.244 (11) ratio. Weak π–π inter­actions between the anthraquinone ring systems [centroid–centroid distance = 3.676 (3) Å], inter­molecular Ag–π inter­actions [*Cg*⋯Ag = 3.405 Å] and C—H⋯π inter­actions between pairs of adjacent mol­ecules are observed.

## Related literature

For the synthesis of 1,8-bis­(2-bromo­eth­oxy)anthraquinone, see: Chen *et al.* (1992 ▶) and of 2-[(1*H*-imidazol-1-yl)meth­yl]pyridine, see: Chiu *et al.* (2005 ▶). For related structures, see: Mahajan *et al.* (2001 ▶, 2002 ▶). For Ag–π inter­actions, see: Mascal *et al.* (2000 ▶).
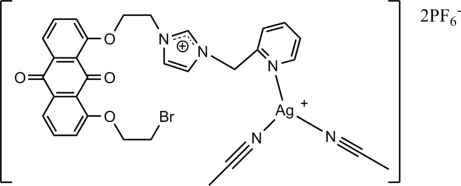


## Experimental

### 

#### Crystal data

[Ag(C_27_H_23_BrN_3_O_4_)(C_2_H_3_N)_2_](PF_6_)_2_
*M* *_r_* = 1013.31
Triclinic, 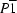

*a* = 7.961 (3) Å
*b* = 12.826 (4) Å
*c* = 18.199 (6) Å
α = 89.034 (14)°
β = 88.278 (12)°
γ = 74.805 (7)°
*V* = 1792.3 (10) Å^3^
*Z* = 2
Mo *K*α radiation
μ = 1.88 mm^−1^
*T* = 116 K
0.20 × 0.18 × 0.16 mm

#### Data collection

Bruker SMART CCD area-detector diffractometer
Absorption correction: multi-scan (*SADABS*; Bruker, 2000 ▶) *T*_min_ = 0.706, *T*_max_ = 0.754
18740 measured reflections
6327 independent reflections
5355 reflections with *I* > 2σ(*I*)
*R*_int_ = 0.062

#### Refinement

*R*[*F* ^2^ > 2σ(*F* ^2^)] = 0.053
*wR*(*F* ^2^) = 0.132
*S* = 1.07
6327 reflections
554 parameters
107 restraints
H-atom parameters constrained
Δρ_max_ = 0.95 e Å^−3^
Δρ_min_ = −1.24 e Å^−3^

### 

Data collection: *SMART* (Bruker, 2000 ▶); cell refinement: *SAINT* (Bruker, 2000 ▶); data reduction: *SAINT*; program(s) used to solve structure: *SHELXS97* (Sheldrick, 2008 ▶); program(s) used to refine structure: *SHELXL97* (Sheldrick, 2008 ▶); molecular graphics: *SHELXTL* (Sheldrick, 2008 ▶); software used to prepare material for publication: *SHELXL97* and *PLATON* (Spek, 2009 ▶).

## Supplementary Material

Crystal structure: contains datablocks global, I. DOI: 10.1107/S1600536810029739/si2276sup1.cif

Structure factors: contains datablocks I. DOI: 10.1107/S1600536810029739/si2276Isup2.hkl

Additional supplementary materials:  crystallographic information; 3D view; checkCIF report

## Figures and Tables

**Table 1:** Selected bond lengths (Å)

Ag1—N4	2.230 (4)
Ag1—N5	2.256 (4)
Ag1—N1	2.284 (4)

**Table 2:** Hydrogen-bond geometry (Å, °) *Cg*1 and *Cg*3 are the centroids of the N2/N3/C7–C9 imidazole rings and C12–C16/C25 anthraquinone rings, respectively.

*D*—H⋯*A*	*D*—H	H⋯*A*	*D*⋯*A*	*D*—H⋯*A*
C26—H26*B*⋯*Cg*3^i^	0.97	2.98	3.845 (5)	148
C31—H31*B*⋯*Cg*1^ii^	0.96	3.38	3.781 (4)	108

Symmetry codes: (i) 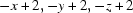; (ii) 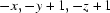.

**Fig. 1.**
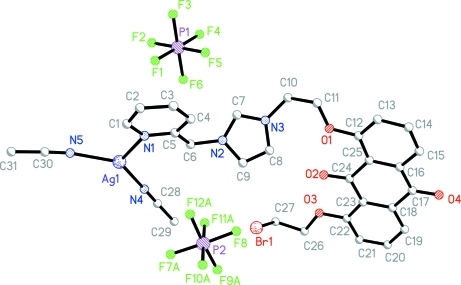
The molecular structure of the title compound, showing 30% probability displacement ellipsoids and the atomic numbering. H atoms and the F7B—F12B disordered positions have been omitted.

**Fig. 2.**
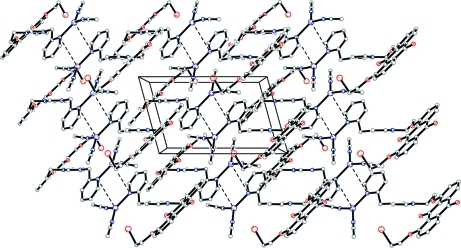
A packing section of the title compound, viewed down the c axis, intermolecular Ag–π interactions shown with dashed lines.

## supplementary crystallographic information

### Comment

Anthraquinone derivatives have attracted much attention due to their optical and photosemiconducting properties (Mahajan *et al.*, 2001, 2002).

The silver(I) atom is bridged by the pyridyl N-atom from one ligand and two acetonitrile molecules and exhibits distorted T shaped coordination (Table 1, Fig. 1). The imidazole rings make dihedral angles with the attached pyridine ring of 61.07 (15)° and anthraquinone ring of 44.80 (10)°. The carbonyl atoms O2 and O4 are almost coplane with the anthraquinone plane, the dihedral angle between the planes of the C23/C24/C25/O2 and C16/C17/C18/O4 fragments is 1.02 (27)°. (Fig. 1).

The crystal structure is stabilized by π–π interactions between the anthraquinone ring systems of the inversion related molecules, with a *Cg*3···*Cg*4^i^ distance of 3.676 (3) Å, [symmetry code i: 1 - *x*, 2 - *y*, 2 - *z*] where *Cg*3 is the C12—C16/C25 ring centroid and *Cg*4 is the C16—C18/C23—C25 ring centroid (Fig. 2), and two adjacent molecules are bridged by Ag···π interactions involving the pyridyl rings: [*Cg*2···Ag1^ii^ = 3.405 Å, (Fig. 2). *Cg*2 is the N1/C1—C5 ring centroid, symmetry code ii: 1 - *x*, 1 - *y*, 1 - *z*]. The present values are thus outside the range of Ag-centroid distances of 2.89–3.37 Å (Mascal *et al.*, 2000). Besides, the structure is further stabilized by C—H···π intermolecular interactions involving the ethoxy carbon C26 and one of the anthraquinone rings [H26B···*Cg*3^iii^ = 2.98 Å, C26—H26B··· *Cg*3^iii^ = 148°, and a weaker contact between one of the acetonitrile donors and the imidazole ring is observed H31B···*Cg*1^iv^ = 3.38 Å, C31—H31B···*Cg*1^iv^ = 108°. *Cg*1 and *Cg*3 are the centroids of the N2/N3/C7—C9 imidazole rings and C12—C16/C25 anthraquinone rings]. Symmetry code for the two interactions, iii: 2 - *x*, 2 - *y*, 2 - *z*; iv: -*x*, 1 - *y*, 1 - *z*.

### Experimental

1,8-Bis(2-bromoethoxy)anthraquinone (Chen *et al.*, 1992) (1.92 g, 10 mmol) was added to a solution of 2-((1*H*-imidazol-1-yl)methyl)pyridine (Chiu *et al.*, 2005) (2.48 g, 20 mmol) in 50 ml of THF. The mixture was refluxed for 48 h. The resulting precipitate was isolated and washed with THF(2 × 5 ml) and was then dissolved in methanol (20 ml). To the aqueous solution was added an excess of NH_4_PF_6_ resulting in a yellow precipitation. The yellow solid was collected and washed with water and Et_2_O and dried under vacuum. The title compound was synthesized from the reaction of a slurry of Ag_2_O (64 mg, 0.275 mmol) in 10 ml of acetonitrile was treated with the yellow soild (213 mg, 0.50 mmol). The mixture was stirred for 12 h with exclusion of light at 323 K until nearly all Ag_2_O was dissolved. The filtrate was concentrated to *ca* 2 ml. Addition of Et_2_O (20 ml) to the filtrate yielded a yellow solid. Yellow single crystals suitable for X-ray diffraction were obtained by recrystallization from acetonitrile.

### Refinement

H atoms were placed in calculated positions with C—H = 0.93–0.97 Å, and refined in riding mode with *U*_iso_(H) = 1.2*U*_eq_(C), and *U*_iso_(H) = 1.5*U*_eq_(C) for the acetonitrile methyl group. In one of the hexafluorophosphate anions five F atoms were found to be disordered over two sites with occupancies 0.756 (11) and 0.244 (11). The disorder was completed with a combination of PLATON (Spek, 2009) and SHELXL97 (Sheldrick, 2008).

### Crystal data

**Table:**

[Ag(C_27_H_23_BrN_3_O_4_)(C_2_H_3_N)_2_](PF_6_)_2_	*Z* = 2
*M**_r_* = 1013.31	*F*(000) = 1004
Triclinic, *P*1	*D*_x_ = 1.878 Mg m^−^^3^
Hall symbol: -P 1	Mo *K*α radiation, λ = 0.71073 Å
*a* = 7.961 (3) Å	Cell parameters from 6148 reflections
*b* = 12.826 (4) Å	θ = 1.6–27.9°
*c* = 18.199 (6) Å	µ = 1.88 mm^−^^1^
α = 89.034 (14)°	*T* = 116 K
β = 88.278 (12)°	Prism, colorless
γ = 74.805 (7)°	0.20 × 0.18 × 0.16 mm
*V* = 1792.3 (10) Å^3^	

### Data collection

**Table:**

Bruker SMART CCD area-detector diffractometer	6327 independent reflections
Radiation source: fine-focus sealed tube	5355 reflections with *I* > 2σ(*I*)
graphite	*R*_int_ = 0.062
Detector resolution: 14.63 pixels mm^-1^	θ_max_ = 25.0°, θ_min_ = 1.1°
ω and φ scans	*h* = −9→9
Absorption correction: multi-scan (*SADABS*; Bruker, 2000)	*k* = −15→15
*T*_min_ = 0.706, *T*_max_ = 0.754	*l* = −21→21
18740 measured reflections	

### Refinement

**Table:**

Refinement on *F*^2^	Secondary atom site location: difference Fourier map
Least-squares matrix: full	Hydrogen site location: inferred from neighbouring sites
*R*[*F*^2^ > 2σ(*F*^2^)] = 0.053	H-atom parameters constrained
*wR*(*F*^2^) = 0.132	*w* = 1/[σ^2^(*F*_o_^2^) + (0.0714*P*)^2^] where *P* = (*F*_o_^2^ + 2*F*_c_^2^)/3
*S* = 1.07	(Δ/σ)_max_ = 0.005
6327 reflections	Δρ_max_ = 0.95 e Å^−^^3^
554 parameters	Δρ_min_ = −1.24 e Å^−^^3^
107 restraints	Extinction correction: *SHELXL97* (Sheldrick, 2008), Fc^*^=kFc[1+0.001xFc^2^λ^3^/sin(2θ)]^-1/4^
Primary atom site location: structure-invariant direct methods	Extinction coefficient: 0.0390 (15)

### Special details

**Table:**

Geometry. All e.s.d.'s (except the e.s.d. in the dihedral angle between two l.s. planes) are estimated using the full covariance matrix. The cell e.s.d.'s are taken into account individually in the estimation of e.s.d.'s in distances, angles and torsion angles; correlations between e.s.d.'s in cell parameters are only used when they are defined by crystal symmetry. An approximate (isotropic) treatment of cell e.s.d.'s is used for estimating e.s.d.'s involving l.s. planes.
Refinement. Refinement of *F*^2^ against ALL reflections. The weighted *R*-factor *wR* and goodness of fit *S* are based on *F*^2^, conventional *R*-factors *R* are based on *F*, with *F* set to zero for negative *F*^2^. The threshold expression of *F*^2^ > σ(*F*^2^) is used only for calculating *R*-factors(gt) *etc*. and is not relevant to the choice of reflections for refinement. *R*-factors based on *F*^2^ are statistically about twice as large as those based on *F*, and *R*- factors based on ALL data will be even larger.

### Fractional atomic coordinates and isotropic or equivalent isotropic displacement parameters (Å^2^)

**Table:**

	*x*	*y*	*z*	*U*_iso_*/*U*_eq_	Occ. (<1)
Ag1	0.81202 (5)	0.46133 (3)	0.492136 (19)	0.02593 (16)	
Br1	1.00740 (8)	0.58237 (5)	0.87898 (3)	0.0448 (2)	
P1	0.80962 (15)	0.90464 (9)	0.40606 (6)	0.0183 (3)	
P2	0.60236 (17)	0.47473 (9)	0.77493 (7)	0.0245 (3)	
F1	0.6881 (4)	0.8274 (2)	0.38733 (15)	0.0309 (6)	
F2	0.9560 (3)	0.8403 (2)	0.34916 (13)	0.0267 (6)	
F3	0.7208 (4)	0.9869 (2)	0.34205 (13)	0.0286 (6)	
F4	0.9307 (4)	0.9829 (2)	0.42395 (14)	0.0297 (6)	
F5	0.6638 (3)	0.9684 (2)	0.46379 (13)	0.0285 (6)	
F6	0.8966 (3)	0.8235 (2)	0.47089 (13)	0.0279 (6)	
F8	0.5481 (5)	0.5903 (2)	0.81166 (17)	0.0503 (9)	
F7A	0.6754 (11)	0.3596 (5)	0.7368 (4)	0.058 (2)	0.756 (11)
F9A	0.4254 (8)	0.4499 (6)	0.7960 (4)	0.0705 (19)	0.756 (11)
F10A	0.6775 (11)	0.4261 (5)	0.8501 (3)	0.0568 (18)	0.756 (11)
F11A	0.7834 (6)	0.5029 (5)	0.7539 (4)	0.0573 (17)	0.756 (11)
F12A	0.5296 (11)	0.5322 (5)	0.6986 (3)	0.062 (2)	0.756 (11)
F7B	0.590 (3)	0.3654 (15)	0.7364 (12)	0.046 (5)	0.244 (11)
F9B	0.3932 (18)	0.5058 (17)	0.7743 (11)	0.058 (5)	0.244 (11)
F10B	0.586 (2)	0.4211 (14)	0.8544 (8)	0.039 (4)	0.244 (11)
F11B	0.8056 (18)	0.439 (2)	0.7781 (11)	0.077 (5)	0.244 (11)
F12B	0.621 (3)	0.5124 (16)	0.6922 (8)	0.049 (5)	0.244 (11)
O1	0.6449 (4)	1.0682 (2)	0.82082 (15)	0.0216 (7)	
O2	0.8220 (5)	0.9164 (3)	0.90418 (16)	0.0362 (9)	
O3	1.0398 (4)	0.7780 (2)	0.98270 (16)	0.0257 (7)	
O4	0.5994 (5)	1.1618 (3)	1.14206 (18)	0.0418 (9)	
N1	0.5981 (5)	0.6198 (3)	0.49115 (18)	0.0183 (8)	
N2	0.7119 (5)	0.7748 (3)	0.64378 (18)	0.0175 (8)	
N3	0.7046 (5)	0.9302 (3)	0.68965 (18)	0.0183 (8)	
N4	0.9818 (5)	0.4191 (3)	0.5892 (2)	0.0250 (9)	
N5	0.8324 (5)	0.3673 (3)	0.3871 (2)	0.0269 (9)	
C1	0.4750 (6)	0.6286 (4)	0.4402 (2)	0.0221 (10)	
H1	0.4891	0.5739	0.4058	0.027*	
C2	0.3295 (6)	0.7149 (4)	0.4368 (2)	0.0234 (10)	
H2	0.2485	0.7193	0.4003	0.028*	
C3	0.3068 (6)	0.7957 (3)	0.4897 (2)	0.0232 (10)	
H3	0.2086	0.8541	0.4898	0.028*	
C4	0.4321 (6)	0.7873 (3)	0.5416 (2)	0.0199 (9)	
H4	0.4192	0.8401	0.5772	0.024*	
C5	0.5769 (6)	0.7000 (3)	0.5405 (2)	0.0176 (9)	
C6	0.7295 (6)	0.6862 (3)	0.5915 (2)	0.0202 (9)	
H6A	0.7429	0.6192	0.6189	0.024*	
H6B	0.8349	0.6799	0.5618	0.024*	
C7	0.7110 (6)	0.8763 (3)	0.6279 (2)	0.0210 (10)	
H7	0.7143	0.9050	0.5808	0.025*	
C8	0.7039 (7)	0.8602 (4)	0.7485 (2)	0.0261 (11)	
H8	0.7017	0.8767	0.7981	0.031*	
C9	0.7073 (6)	0.7634 (4)	0.7195 (2)	0.0240 (10)	
H9	0.7065	0.7006	0.7456	0.029*	
C10	0.7066 (7)	1.0449 (3)	0.6937 (2)	0.0241 (10)	
H10A	0.6794	1.0784	0.6458	0.029*	
H10B	0.8232	1.0484	0.7051	0.029*	
C11	0.5801 (6)	1.1092 (3)	0.7502 (2)	0.0237 (10)	
H11A	0.5744	1.1855	0.7456	0.028*	
H11B	0.4644	1.0997	0.7439	0.028*	
C12	0.5707 (6)	1.1236 (3)	0.8828 (2)	0.0196 (9)	
C13	0.4385 (6)	1.2174 (3)	0.8799 (3)	0.0246 (10)	
H13	0.3939	1.2440	0.8347	0.030*	
C14	0.3706 (6)	1.2731 (4)	0.9440 (3)	0.0286 (11)	
H14	0.2796	1.3356	0.9415	0.034*	
C15	0.4388 (6)	1.2352 (4)	1.0106 (3)	0.0299 (11)	
H15	0.3952	1.2729	1.0532	0.036*	
C16	0.5722 (6)	1.1412 (4)	1.0147 (2)	0.0230 (10)	
C17	0.6479 (6)	1.1053 (4)	1.0879 (2)	0.0277 (11)	
C18	0.7802 (6)	1.0009 (4)	1.0945 (2)	0.0245 (10)	
C19	0.8386 (7)	0.9673 (4)	1.1646 (2)	0.0303 (12)	
H19	0.7945	1.0098	1.2052	0.036*	
C20	0.9628 (7)	0.8701 (4)	1.1732 (2)	0.0310 (12)	
H20	1.0008	0.8475	1.2201	0.037*	
C21	1.0319 (7)	0.8060 (4)	1.1136 (2)	0.0279 (11)	
H21	1.1157	0.7410	1.1204	0.033*	
C22	0.9738 (6)	0.8400 (4)	1.0423 (2)	0.0206 (10)	
C23	0.8463 (6)	0.9375 (3)	1.0320 (2)	0.0206 (10)	
C24	0.7757 (6)	0.9741 (3)	0.9574 (2)	0.0198 (10)	
C25	0.6409 (6)	1.0806 (3)	0.9513 (2)	0.0193 (9)	
C26	1.1559 (6)	0.6745 (4)	0.9962 (2)	0.0276 (11)	
H26A	1.0995	0.6313	1.0277	0.033*	
H26B	1.2590	0.6824	1.0203	0.033*	
C27	1.2049 (7)	0.6212 (4)	0.9225 (3)	0.0335 (12)	
H27A	1.2995	0.5566	0.9285	0.040*	
H27B	1.2456	0.6700	0.8895	0.040*	
C28	1.0521 (6)	0.3993 (3)	0.6423 (3)	0.0216 (10)	
C29	1.1464 (7)	0.3739 (4)	0.7117 (3)	0.0310 (11)	
H29A	1.2696	0.3538	0.7012	0.046*	
H29B	1.1138	0.3150	0.7363	0.046*	
H29C	1.1172	0.4362	0.7427	0.046*	
C30	0.8236 (6)	0.3136 (4)	0.3397 (3)	0.0266 (11)	
C31	0.8093 (7)	0.2415 (4)	0.2798 (3)	0.0383 (13)	
H31A	0.8302	0.1688	0.2983	0.057*	
H31B	0.8938	0.2447	0.2416	0.057*	
H31C	0.6945	0.2639	0.2603	0.057*	

### Atomic displacement parameters (Å^2^)

**Table:**

	*U*^11^	*U*^22^	*U*^33^	*U*^12^	*U*^13^	*U*^23^
Ag1	0.0270 (2)	0.0223 (2)	0.0255 (2)	−0.00068 (16)	−0.00450 (15)	−0.00217 (15)
Br1	0.0453 (4)	0.0449 (4)	0.0436 (4)	−0.0092 (3)	−0.0102 (3)	−0.0097 (3)
P1	0.0207 (6)	0.0191 (6)	0.0130 (6)	−0.0012 (5)	−0.0021 (4)	0.0012 (5)
P2	0.0289 (7)	0.0217 (6)	0.0232 (6)	−0.0068 (5)	−0.0067 (5)	0.0027 (5)
F1	0.0292 (16)	0.0306 (15)	0.0346 (15)	−0.0105 (13)	−0.0048 (12)	0.0012 (12)
F2	0.0294 (15)	0.0287 (14)	0.0195 (13)	−0.0040 (12)	0.0066 (11)	−0.0033 (11)
F3	0.0364 (16)	0.0287 (15)	0.0169 (13)	−0.0014 (12)	−0.0066 (11)	0.0089 (11)
F4	0.0333 (16)	0.0279 (14)	0.0301 (15)	−0.0107 (13)	−0.0076 (12)	−0.0040 (12)
F5	0.0312 (16)	0.0242 (14)	0.0214 (13)	0.0075 (12)	0.0056 (11)	0.0005 (11)
F6	0.0295 (15)	0.0271 (14)	0.0188 (13)	0.0073 (12)	−0.0032 (11)	0.0065 (11)
F8	0.075 (3)	0.0322 (17)	0.0439 (19)	−0.0153 (17)	0.0184 (17)	−0.0086 (14)
F7A	0.101 (5)	0.025 (2)	0.048 (3)	−0.019 (3)	0.016 (4)	−0.014 (2)
F9A	0.052 (3)	0.069 (4)	0.104 (4)	−0.041 (3)	0.001 (3)	0.003 (4)
F10A	0.081 (5)	0.047 (3)	0.039 (3)	−0.008 (3)	−0.029 (3)	0.017 (2)
F11A	0.031 (3)	0.039 (3)	0.101 (4)	−0.010 (2)	0.021 (2)	−0.008 (3)
F12A	0.100 (5)	0.045 (3)	0.030 (3)	0.003 (3)	−0.032 (3)	0.003 (2)
F7B	0.077 (9)	0.025 (6)	0.040 (7)	−0.020 (7)	−0.004 (8)	−0.006 (5)
F9B	0.039 (7)	0.064 (9)	0.073 (8)	−0.015 (7)	−0.009 (6)	−0.010 (7)
F10B	0.047 (7)	0.043 (6)	0.031 (6)	−0.017 (6)	−0.002 (6)	0.007 (5)
F11B	0.056 (7)	0.086 (9)	0.084 (8)	−0.009 (7)	−0.007 (6)	0.002 (7)
F12B	0.063 (9)	0.042 (7)	0.039 (7)	−0.014 (7)	0.014 (7)	0.019 (6)
O1	0.0293 (18)	0.0182 (15)	0.0139 (15)	0.0003 (13)	−0.0081 (13)	−0.0001 (12)
O2	0.056 (2)	0.0275 (18)	0.0143 (17)	0.0098 (17)	−0.0124 (16)	−0.0040 (14)
O3	0.034 (2)	0.0247 (17)	0.0147 (15)	−0.0007 (14)	−0.0071 (13)	0.0042 (13)
O4	0.055 (3)	0.049 (2)	0.0219 (19)	−0.014 (2)	0.0068 (17)	−0.0133 (17)
N1	0.023 (2)	0.0153 (18)	0.0153 (18)	−0.0027 (15)	−0.0030 (15)	0.0011 (15)
N2	0.021 (2)	0.0151 (18)	0.0160 (18)	−0.0034 (15)	−0.0044 (15)	0.0016 (15)
N3	0.026 (2)	0.0179 (18)	0.0122 (18)	−0.0070 (16)	−0.0040 (15)	0.0020 (15)
N4	0.024 (2)	0.0193 (19)	0.029 (2)	−0.0021 (17)	−0.0014 (18)	0.0023 (17)
N5	0.029 (2)	0.029 (2)	0.022 (2)	−0.0064 (18)	−0.0064 (17)	−0.0001 (18)
C1	0.026 (3)	0.022 (2)	0.020 (2)	−0.009 (2)	−0.0059 (19)	0.0000 (19)
C2	0.022 (2)	0.027 (2)	0.023 (2)	−0.010 (2)	−0.0094 (19)	0.004 (2)
C3	0.019 (2)	0.019 (2)	0.031 (3)	−0.0040 (19)	−0.0051 (19)	0.0036 (19)
C4	0.023 (2)	0.016 (2)	0.021 (2)	−0.0057 (18)	−0.0014 (18)	−0.0008 (18)
C5	0.021 (2)	0.016 (2)	0.017 (2)	−0.0072 (18)	−0.0028 (17)	0.0023 (17)
C6	0.024 (2)	0.017 (2)	0.018 (2)	−0.0010 (18)	−0.0066 (18)	−0.0029 (18)
C7	0.028 (3)	0.023 (2)	0.012 (2)	−0.006 (2)	−0.0053 (18)	0.0029 (18)
C8	0.042 (3)	0.025 (2)	0.014 (2)	−0.013 (2)	−0.004 (2)	0.0014 (19)
C9	0.038 (3)	0.021 (2)	0.014 (2)	−0.010 (2)	−0.0018 (19)	0.0009 (18)
C10	0.043 (3)	0.017 (2)	0.015 (2)	−0.012 (2)	−0.004 (2)	0.0027 (18)
C11	0.035 (3)	0.017 (2)	0.018 (2)	−0.005 (2)	−0.0090 (19)	0.0026 (18)
C12	0.020 (2)	0.022 (2)	0.020 (2)	−0.0101 (19)	−0.0017 (18)	−0.0020 (18)
C13	0.025 (3)	0.022 (2)	0.027 (2)	−0.005 (2)	−0.003 (2)	0.001 (2)
C14	0.026 (3)	0.021 (2)	0.038 (3)	−0.005 (2)	0.002 (2)	−0.007 (2)
C15	0.031 (3)	0.028 (3)	0.031 (3)	−0.009 (2)	0.012 (2)	−0.010 (2)
C16	0.027 (3)	0.025 (2)	0.022 (2)	−0.014 (2)	0.0038 (19)	−0.0032 (19)
C17	0.033 (3)	0.033 (3)	0.021 (2)	−0.018 (2)	0.006 (2)	−0.007 (2)
C18	0.031 (3)	0.036 (3)	0.014 (2)	−0.022 (2)	0.0004 (19)	0.002 (2)
C19	0.044 (3)	0.043 (3)	0.012 (2)	−0.028 (3)	0.002 (2)	−0.004 (2)
C20	0.047 (3)	0.045 (3)	0.012 (2)	−0.032 (3)	−0.011 (2)	0.009 (2)
C21	0.034 (3)	0.034 (3)	0.021 (2)	−0.017 (2)	−0.011 (2)	0.008 (2)
C22	0.025 (3)	0.029 (2)	0.013 (2)	−0.016 (2)	−0.0044 (18)	0.0019 (18)
C23	0.026 (2)	0.025 (2)	0.016 (2)	−0.015 (2)	−0.0014 (18)	−0.0019 (18)
C24	0.026 (3)	0.023 (2)	0.013 (2)	−0.011 (2)	−0.0039 (18)	0.0018 (19)
C25	0.024 (2)	0.019 (2)	0.016 (2)	−0.0082 (19)	0.0023 (18)	−0.0031 (18)
C26	0.030 (3)	0.025 (2)	0.027 (3)	−0.006 (2)	−0.007 (2)	0.006 (2)
C27	0.029 (3)	0.034 (3)	0.035 (3)	−0.003 (2)	−0.002 (2)	−0.002 (2)
C28	0.020 (2)	0.016 (2)	0.027 (3)	−0.0017 (18)	0.001 (2)	−0.0034 (19)
C29	0.034 (3)	0.027 (3)	0.030 (3)	−0.003 (2)	−0.011 (2)	−0.002 (2)
C30	0.025 (3)	0.029 (3)	0.026 (3)	−0.007 (2)	−0.004 (2)	0.003 (2)
C31	0.048 (4)	0.033 (3)	0.032 (3)	−0.006 (3)	−0.006 (2)	−0.011 (2)

### Geometric parameters (Å, °)

**Table:**

Ag1—N4	2.230 (4)	C6—H6A	0.9700
Ag1—N5	2.256 (4)	C6—H6B	0.9700
Ag1—N1	2.284 (4)	C7—H7	0.9300
Br1—C27	1.961 (5)	C8—C9	1.351 (6)
P1—F2	1.601 (3)	C8—H8	0.9300
P1—F1	1.602 (3)	C9—H9	0.9300
P1—F5	1.606 (3)	C10—C11	1.511 (6)
P1—F4	1.606 (3)	C10—H10A	0.9700
P1—F6	1.608 (2)	C10—H10B	0.9700
P1—F3	1.609 (3)	C11—H11A	0.9700
P2—F9A	1.560 (5)	C11—H11B	0.9700
P2—F11B	1.564 (14)	C12—C13	1.376 (6)
P2—F10A	1.563 (5)	C12—C25	1.424 (6)
P2—F7A	1.600 (6)	C13—C14	1.397 (6)
P2—F8	1.585 (3)	C13—H13	0.9300
P2—F11A	1.609 (5)	C14—C15	1.372 (7)
P2—F12A	1.612 (5)	C14—H14	0.9300
P2—F7B	1.607 (15)	C15—C16	1.385 (7)
P2—F9B	1.608 (14)	C15—H15	0.9300
P2—F10B	1.608 (13)	C16—C25	1.415 (6)
P2—F12B	1.588 (14)	C16—C17	1.494 (6)
O1—C12	1.376 (5)	C17—C18	1.476 (7)
O1—C11	1.438 (5)	C18—C19	1.394 (6)
O2—C24	1.217 (5)	C18—C23	1.414 (6)
O3—C22	1.364 (5)	C19—C20	1.382 (7)
O3—C26	1.427 (5)	C19—H19	0.9300
O4—C17	1.225 (5)	C20—C21	1.382 (7)
N1—C5	1.351 (5)	C20—H20	0.9300
N1—C1	1.351 (5)	C21—C22	1.415 (6)
N2—C7	1.326 (5)	C21—H21	0.9300
N2—C9	1.384 (5)	C22—C23	1.403 (6)
N2—C6	1.471 (5)	C23—C24	1.506 (6)
N3—C7	1.321 (5)	C24—C25	1.505 (6)
N3—C8	1.386 (5)	C26—C27	1.510 (7)
N3—C10	1.479 (5)	C26—H26A	0.9700
N4—C28	1.122 (6)	C26—H26B	0.9700
N5—C30	1.129 (6)	C27—H27A	0.9700
C1—C2	1.379 (6)	C27—H27B	0.9700
C1—H1	0.9300	C28—C29	1.476 (6)
C2—C3	1.398 (6)	C29—H29A	0.9600
C2—H2	0.9300	C29—H29B	0.9600
C3—C4	1.377 (6)	C29—H29C	0.9600
C3—H3	0.9300	C30—C31	1.467 (7)
C4—C5	1.381 (6)	C31—H31A	0.9600
C4—H4	0.9300	C31—H31B	0.9600
C5—C6	1.522 (6)	C31—H31C	0.9600
			
N4—Ag1—N5	127.22 (14)	N2—C6—C5	114.4 (3)
N4—Ag1—N1	119.44 (13)	N2—C6—H6A	108.6
N5—Ag1—N1	113.27 (13)	C5—C6—H6A	108.6
F2—P1—F1	90.22 (15)	N2—C6—H6B	108.6
F2—P1—F5	179.42 (16)	C5—C6—H6B	108.6
F1—P1—F5	89.84 (15)	H6A—C6—H6B	107.6
F2—P1—F4	89.66 (15)	N3—C7—N2	109.3 (4)
F1—P1—F4	179.30 (16)	N3—C7—H7	125.4
F5—P1—F4	90.28 (15)	N2—C7—H7	125.4
F2—P1—F6	90.03 (14)	C9—C8—N3	106.5 (4)
F1—P1—F6	90.27 (15)	C9—C8—H8	126.8
F5—P1—F6	89.39 (14)	N3—C8—H8	126.8
F4—P1—F6	90.42 (15)	C8—C9—N2	107.4 (4)
F2—P1—F3	90.77 (14)	C8—C9—H9	126.3
F1—P1—F3	89.85 (15)	N2—C9—H9	126.3
F5—P1—F3	89.81 (14)	N3—C10—C11	114.1 (4)
F4—P1—F3	89.46 (14)	N3—C10—H10A	108.7
F6—P1—F3	179.18 (16)	C11—C10—H10A	108.7
F9A—P2—F11B	148.0 (9)	N3—C10—H10B	108.7
F9A—P2—F10A	90.0 (4)	C11—C10—H10B	108.7
F11B—P2—F10A	64.6 (8)	H10A—C10—H10B	107.6
F9A—P2—F7A	92.0 (3)	O1—C11—C10	106.2 (4)
F11B—P2—F7A	70.3 (8)	O1—C11—H11A	110.5
F10A—P2—F7A	90.5 (4)	C10—C11—H11A	110.5
F9A—P2—F8	93.4 (3)	O1—C11—H11B	110.5
F11B—P2—F8	104.5 (9)	C10—C11—H11B	110.5
F10A—P2—F8	88.7 (3)	H11A—C11—H11B	108.7
F7A—P2—F8	174.5 (4)	C13—C12—O1	122.7 (4)
F9A—P2—F11A	178.8 (3)	C13—C12—C25	120.8 (4)
F10A—P2—F11A	90.0 (3)	O1—C12—C25	116.5 (4)
F7A—P2—F11A	89.2 (3)	C12—C13—C14	120.9 (4)
F8—P2—F11A	85.4 (2)	C12—C13—H13	119.5
F9A—P2—F12A	92.6 (4)	C14—C13—H13	119.5
F11B—P2—F12A	114.0 (8)	C15—C14—C13	119.6 (5)
F10A—P2—F12A	176.0 (3)	C15—C14—H14	120.2
F7A—P2—F12A	92.5 (4)	C13—C14—H14	120.2
F8—P2—F12A	88.1 (3)	C14—C15—C16	120.4 (4)
F11A—P2—F12A	87.4 (3)	C14—C15—H15	119.8
F9A—P2—F7B	69.7 (8)	C16—C15—H15	119.8
F11B—P2—F7B	94.2 (10)	C15—C16—C25	121.7 (4)
F10A—P2—F7B	98.9 (9)	C15—C16—C17	118.6 (4)
F8—P2—F7B	161.3 (8)	C25—C16—C17	119.7 (4)
F11A—P2—F7B	111.5 (8)	O4—C17—C18	120.4 (4)
F12A—P2—F7B	84.8 (9)	O4—C17—C16	120.2 (5)
F11B—P2—F9B	177.1 (10)	C18—C17—C16	119.5 (4)
F10A—P2—F9B	113.1 (7)	C19—C18—C23	121.1 (5)
F7A—P2—F9B	108.4 (7)	C19—C18—C17	117.5 (4)
F8—P2—F9B	76.8 (7)	C23—C18—C17	121.4 (4)
F11A—P2—F9B	150.1 (8)	C20—C19—C18	119.4 (4)
F12A—P2—F9B	68.5 (7)	C20—C19—H19	120.3
F7B—P2—F9B	84.4 (9)	C18—C19—H19	120.3
F9A—P2—F10B	62.8 (6)	C19—C20—C21	121.5 (4)
F11B—P2—F10B	90.8 (9)	C19—C20—H20	119.3
F7A—P2—F10B	92.6 (7)	C21—C20—H20	119.3
F8—P2—F10B	89.2 (6)	C20—C21—C22	119.4 (5)
F11A—P2—F10B	117.2 (7)	C20—C21—H21	120.3
F12A—P2—F10B	155.0 (7)	C22—C21—H21	120.3
F7B—P2—F10B	90.0 (11)	O3—C22—C23	119.2 (4)
F9B—P2—F10B	86.7 (9)	O3—C22—C21	120.5 (4)
F9A—P2—F12B	115.1 (7)	C23—C22—C21	120.3 (4)
F11B—P2—F12B	88.7 (10)	C22—C23—C18	118.3 (4)
F10A—P2—F12B	153.3 (7)	C22—C23—C24	121.9 (4)
F7A—P2—F12B	80.1 (8)	C18—C23—C24	119.8 (4)
F8—P2—F12B	98.3 (7)	O2—C24—C25	121.3 (4)
F11A—P2—F12B	65.1 (7)	O2—C24—C23	120.4 (4)
F7B—P2—F12B	82.6 (11)	C25—C24—C23	118.3 (4)
F9B—P2—F12B	93.7 (9)	C16—C25—C12	116.6 (4)
F10B—P2—F12B	172.5 (10)	C16—C25—C24	121.0 (4)
C12—O1—C11	118.6 (3)	C12—C25—C24	122.4 (4)
C22—O3—C26	117.3 (3)	O3—C26—C27	107.0 (4)
C5—N1—C1	118.0 (4)	O3—C26—H26A	110.3
C5—N1—Ag1	125.6 (3)	C27—C26—H26A	110.3
C1—N1—Ag1	116.1 (3)	O3—C26—H26B	110.3
C7—N2—C9	108.1 (3)	C27—C26—H26B	110.3
C7—N2—C6	126.9 (4)	H26A—C26—H26B	108.6
C9—N2—C6	124.8 (3)	C26—C27—Br1	111.7 (3)
C7—N3—C8	108.7 (4)	C26—C27—H27A	109.3
C7—N3—C10	124.6 (3)	Br1—C27—H27A	109.3
C8—N3—C10	126.6 (3)	C26—C27—H27B	109.3
C28—N4—Ag1	173.0 (4)	Br1—C27—H27B	109.3
C30—N5—Ag1	169.3 (4)	H27A—C27—H27B	107.9
N1—C1—C2	123.0 (4)	N4—C28—C29	179.2 (5)
N1—C1—H1	118.5	C28—C29—H29A	109.5
C2—C1—H1	118.5	C28—C29—H29B	109.5
C1—C2—C3	118.3 (4)	H29A—C29—H29B	109.5
C1—C2—H2	120.8	C28—C29—H29C	109.5
C3—C2—H2	120.8	H29A—C29—H29C	109.5
C4—C3—C2	118.9 (4)	H29B—C29—H29C	109.5
C4—C3—H3	120.5	N5—C30—C31	178.0 (5)
C2—C3—H3	120.5	C30—C31—H31A	109.5
C3—C4—C5	119.7 (4)	C30—C31—H31B	109.5
C3—C4—H4	120.2	H31A—C31—H31B	109.5
C5—C4—H4	120.2	C30—C31—H31C	109.5
N1—C5—C4	122.1 (4)	H31A—C31—H31C	109.5
N1—C5—C6	113.6 (4)	H31B—C31—H31C	109.5
C4—C5—C6	124.3 (4)		
			
N4—Ag1—N1—C5	9.4 (4)	C15—C16—C17—O4	−4.4 (7)
N5—Ag1—N1—C5	−167.7 (3)	C25—C16—C17—O4	173.9 (4)
N4—Ag1—N1—C1	−164.6 (3)	C15—C16—C17—C18	175.1 (4)
N5—Ag1—N1—C1	18.2 (3)	C25—C16—C17—C18	−6.6 (6)
N5—Ag1—N4—C28	−129 (3)	O4—C17—C18—C19	3.7 (7)
N1—Ag1—N4—C28	55 (3)	C16—C17—C18—C19	−175.8 (4)
N4—Ag1—N5—C30	96 (2)	O4—C17—C18—C23	−175.9 (4)
N1—Ag1—N5—C30	−87 (2)	C16—C17—C18—C23	4.6 (6)
C5—N1—C1—C2	−0.4 (6)	C23—C18—C19—C20	−0.2 (7)
Ag1—N1—C1—C2	174.1 (3)	C17—C18—C19—C20	−179.8 (4)
N1—C1—C2—C3	−1.5 (7)	C18—C19—C20—C21	0.7 (7)
C1—C2—C3—C4	1.6 (6)	C19—C20—C21—C22	−0.3 (7)
C2—C3—C4—C5	0.1 (6)	C26—O3—C22—C23	−173.8 (4)
C1—N1—C5—C4	2.2 (6)	C26—O3—C22—C21	5.6 (6)
Ag1—N1—C5—C4	−171.7 (3)	C20—C21—C22—O3	−179.9 (4)
C1—N1—C5—C6	−175.5 (4)	C20—C21—C22—C23	−0.5 (6)
Ag1—N1—C5—C6	10.5 (5)	O3—C22—C23—C18	−179.7 (4)
C3—C4—C5—N1	−2.1 (6)	C21—C22—C23—C18	0.9 (6)
C3—C4—C5—C6	175.4 (4)	O3—C22—C23—C24	2.1 (6)
C7—N2—C6—C5	−66.6 (6)	C21—C22—C23—C24	−177.3 (4)
C9—N2—C6—C5	118.8 (4)	C19—C18—C23—C22	−0.6 (6)
N1—C5—C6—N2	177.8 (4)	C17—C18—C23—C22	179.0 (4)
C4—C5—C6—N2	0.1 (6)	C19—C18—C23—C24	177.7 (4)
C8—N3—C7—N2	0.9 (5)	C17—C18—C23—C24	−2.7 (6)
C10—N3—C7—N2	178.2 (4)	C22—C23—C24—O2	3.8 (7)
C9—N2—C7—N3	−0.5 (5)	C18—C23—C24—O2	−174.5 (4)
C6—N2—C7—N3	−175.8 (4)	C22—C23—C24—C25	−179.1 (4)
C7—N3—C8—C9	−1.0 (5)	C18—C23—C24—C25	2.6 (6)
C10—N3—C8—C9	−178.2 (4)	C15—C16—C25—C12	2.5 (6)
N3—C8—C9—N2	0.7 (5)	C17—C16—C25—C12	−175.8 (4)
C7—N2—C9—C8	−0.1 (5)	C15—C16—C25—C24	−175.0 (4)
C6—N2—C9—C8	175.3 (4)	C17—C16—C25—C24	6.7 (6)
C7—N3—C10—C11	137.8 (4)	C13—C12—C25—C16	−2.2 (6)
C8—N3—C10—C11	−45.3 (6)	O1—C12—C25—C16	176.1 (4)
C12—O1—C11—C10	169.7 (3)	C13—C12—C25—C24	175.2 (4)
N3—C10—C11—O1	68.5 (5)	O1—C12—C25—C24	−6.5 (6)
C11—O1—C12—C13	−0.9 (6)	O2—C24—C25—C16	172.3 (4)
C11—O1—C12—C25	−179.2 (4)	C23—C24—C25—C16	−4.8 (6)
O1—C12—C13—C14	−177.9 (4)	O2—C24—C25—C12	−5.0 (7)
C25—C12—C13—C14	0.4 (7)	C23—C24—C25—C12	177.9 (4)
C12—C13—C14—C15	1.4 (7)	C22—O3—C26—C27	178.1 (4)
C13—C14—C15—C16	−1.1 (7)	O3—C26—C27—Br1	−69.2 (4)
C14—C15—C16—C25	−0.8 (7)	Ag1—N4—C28—C29	−146 (40)
C14—C15—C16—C17	177.5 (4)	Ag1—N5—C30—C31	−17 (17)

### Hydrogen-bond geometry (Å, °)

**Table:**

*Cg*1 and *Cg*3 are the centroids of the N2/N3/C7–C9 imidazole rings and C12–C16/C25 anthraquinone rings, respectively.

**Table:**

*D*—H···*A*	*D*—H	H···*A*	*D*···*A*	*D*—H···*A*
C26—H26B···Cg3^i^	0.97	2.98	3.845 (5)	148
C31—H31B···Cg1^ii^	0.96	3.38	3.781 (4)	108

Symmetry codes: (i) −*x*+2, −*y*+2, −*z*+2; (ii) −*x*, −*y*+1, −*z*+1.
